# Influence of Social Media on Corporate Communication Social Responsibility Under Entrepreneurial Psychology

**DOI:** 10.3389/fpsyg.2022.870373

**Published:** 2022-06-16

**Authors:** Mufei Cai

**Affiliations:** Marketing Department, Amsterdam Business School, University of Amsterdam, Amsterdam, Netherlands

**Keywords:** entrepreneurial psychology, social media, impact of CSR communication, CSR scores, social responsibility

## Abstract

The research intends to improve the optimization of social media’s nature of corporate social responsibility (CSR) and standardize the influence mechanism of social media. First, the research analyzes the concepts of social media, CSR, and corporate reputation from the perspective of entrepreneurial psychology and expounds on the influencing factors of CSR scores from a macro perspective. Second, the mechanism of social media’s role in CSR is briefly discussed. On this basis, it is found that the most intuitive manifestation of social media platforms affecting the communication of CSR is the impact on the score of CSR. Three hypotheses are proposed, namely, (1) social media platforms, such as “WeChat,” have a greater impact on the communication of CSR; (2) social media is positively correlated with the increase in CSR scores; and (3) user reading and liking are positively correlated with the increase in CSR scores. Finally, 78 listed companies related to the mobile communication industry are selected as samples for the questionnaire survey and statistical analysis, and the hypothesis is demonstrated. The results demonstrate that the hypothesis that the social media platform “WeChat” has an impact on the communication of CSR is valid, and the hypothesis that the open state of “social media” is positively correlated with the increase in CSR scores is not valid. It is assumed that the number of user readings and likes is positively correlated with the increase in CSR score, which is valid under a limited sample. It is concluded that the WeChat platform has the best effect on the communication of CSR and can provide the impetus for the improvement of corporate reputation. The opening of “social media” is not directly related to the improvement of CSR scores. The correct operation of “social media” will have an impact on the communication of CSR. The correlation between the number of users reading and liking on “social media” and the increase in CSR score is not significant, as only the number of “likes” on social media related to shareholders is significantly associated with an increase in CSR score. The linear regression coefficient between the number of likes and the increase in CSR score is less than 0.05. There is a positive correlation between user reads, likes, and increases in CSR scores. This research helps enterprises to effectively fulfill their social responsibilities and improve the efficiency of CSR. This makes up for the lack of social media’s influence mechanism on the nature of CSR. It innovatively explores the impact of social media on CSR from the perspective of entrepreneurial psychology and provides some ideas for entrepreneurs and enterprises to create CSR and CSR value.

## Introduction

Economic development increasingly promotes social development. However, while enterprises enjoy the opportunities brought by social development, they are also accompanied by crises such as food safety, safety problems, fraud problems, and reputation problems (Lu, 2019). In the past, companies issued social responsibility reports every year to undertake corporate social responsibility (CSR). The impact of CSR communication refers to the process and behavior of sending or communicating the relevant practices and information of CSR to stakeholders and to gain further understanding and recognition of its CSR from the outside world. CSR score means the systematic evaluation of CSR, which can comprehensively and objectively reflect the performance of CSR (Zhang, 2020). At present, the development of the Internet has led to the emergence of social media such as Weibo and WeChat. With its own advantages, social media has quickly been favored by all walks of life (Gao and Liang, 2020). Corporates also use social media to undertake CSR and explore the relationship between current customers and other stakeholders, thereby enhancing their reputation (Guo, 2019). Entrepreneurial psychology is a science that studies people’s psychological activities and behavioral laws in the process of starting a business. It is a comprehensively applied subject and a branch of the psychological characteristics of individual personality. After learning, individuals use the general laws of psychology to solve psychological problems encountered in the process of starting a business. A good state of entrepreneurial spirit can enable entrepreneurs to achieve their desired goals more effectively.

There are some studies on CSR communication in recent years. Zhu et al. (2020) constructed a knowledge communication model on the two-tier coupling network of corporate social media and employee offline interaction and discussed the influencing factors of corporate social media on CSR communication (Zhu et al., 2020). Hao and Li (2018) constructed an index system from three aspects, namely, managerial characteristics, enterprise characteristics, and social supervision characteristics, and established an ordered profit model to study the influencing factors of CSR fulfillment (Hao and Li, 2018). Mahmood et al. (2021) studied the influencing factors and economic consequences of CSR and found that CSR is conducive to the improvement of corporate performance (Mahmood et al., 2021). Liu X. P. et al. (2020) explored the influencing factors of consumer willingness to continue to participate in CSR activities, such as “CSR-CA,” “social existence,” and “self-construction,” with the help of relevant theoretical perspectives in social media and other fields (Liu X. P. et al., 2020). Hayes and Carr (2021) argued that users’ feedback to enterprises on social media is unknown. They also studied the impact of positive and negative social comments on CSR and the response of enterprises (Hayes and Carr, 2021). He et al. (2021) established a preliminary framework for the factors that affect consumers’ response to illegal brands (i.e., Facebook) and resist brands (i.e., advertisers) and discussed the irresponsibility and CSR of traditional enterprises (He et al., 2021). Through literature review, previous studies on social media and CSR have been conducted from one aspect, many of which focus on customer consumption. However, the influence mechanism of social media on the essence of CSR is insufficient. To sum up, research from the perspective of entrepreneurial psychology is also an innovation. Based on this, the impact of social media on CSR is studied through analyses, assumptions, and arguments.

First, this study analyzes the concepts of social media, CSR, and corporate reputation under the background of entrepreneurial psychology and expounds on the influencing factors of CSR scores from a macro perspective. Second, the mechanism of social media’s role in CSR is briefly discussed. The most intuitive manifestation of social media platforms affecting the communication of CSR is the impact on the score of CSR, and three hypotheses are proposed. Finally, 78 listed companies related to the mobile communication industry are selected as samples for the questionnaire survey and statistical analysis, and the hypothesis is demonstrated. The research results obtained have a profound reference significance for enterprises to create CSR and CSR value.

## Corporate Social Responsibility From the Perspective of Entrepreneurial Psychology

### Theoretical Analysis of Entrepreneurial Psychology

Entrepreneurial psychology is a science that studies people’s psychological activities and their behavioral laws in the process of entrepreneurship (Luo, 2019). It is a comprehensive application discipline. Entrepreneurial psychology is a branch of psychological characteristics of individual personality. Individuals solve the psychological problems encountered in the process of entrepreneurship by using the general laws of psychology after learning. Having a good entrepreneurial psychological state can make entrepreneurs more effectively achieve the expected goals.

“Entrepreneurial psychology” is also a course in the entrepreneurial discipline system. It mainly trains students’ psychological qualities, such as tenacity, adaptability, and cooperation, and overcomes the behavioral obstacles, such as personality obstacles and changeable goals, in the process of entrepreneurship. Its main content is personality characteristics and psychological development of college students; analysis of entrepreneurs’ psychological quality and entrepreneurial process; and psychological coping in the period of success and frustration (Qiang and Du, 2019). School innovation and entrepreneurship education can promote students to form correct value according to the relevant content of entrepreneurship psychology, so that students’ psychological energy in the entrepreneurial and “quasi-entrepreneurship” can be correctly released.

### Definition Analysis of Social Media

Social medium is a virtual community and a network platform for people to create, share, and exchange experience, allowing users to present texts, images, music, and videos (Yan, 2020). Social medium is one of the most popular media in Internet media with its excellent characteristics and becomes an important force in communication (Wang et al., 2020). Social media are widely used in library management, government management, teaching management, and other fields (Chen et al., 2020).

**FIGURE 1:**
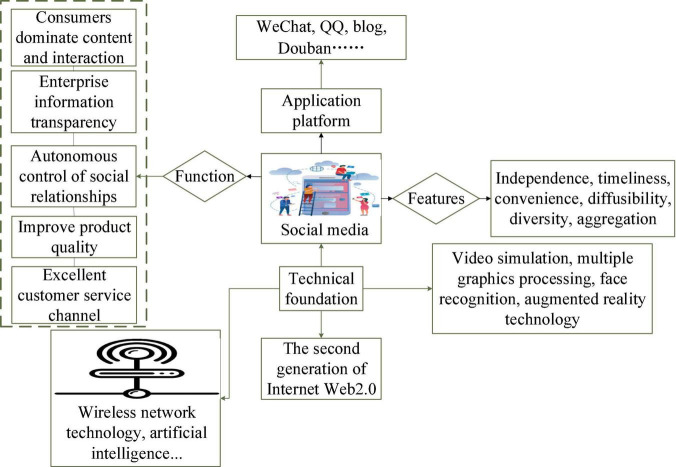
Architecture of social media.

Social media originate from the development of Web 2.0 (Wang, 2020). The development of the Internet and mobile platform technology makes the social function of social media increasingly prominent and becomes an important platform for communication between individuals and organizations. The architecture of social media is shown in Figure 1.

### Analysis of Corporate Social Responsibility

Corporate social responsibility refers to the responsibility of enterprises to other stakeholders and social public interests, while they are legally operating, creating profits, and taking responsibility for shareholders (Fan et al., 2020). The earliest systematic definition of CSR is proposed by Bowen (1953). He believed that the businessman’s social responsibility is to formulate policies and make decisions according to the social goals and values.

Corporate social responsibility is the unity of legal obligation and moral obligation. From an institutional perspective, CSR is a unity of coexistence of formal and informal institutions (Tian and Suo, 2020). In fact, CSR includes charity, public welfare, and other narrow sense contents, and the performance of CSR has nothing to do with the nature of ownership of enterprises. It also has nothing to do with the specific stage of the development of enterprises. From the perspective of social citizens, enterprises should fulfill the responsibilities of all stakeholders. At present, it becomes a consensus that CSR can create value for enterprises. From the perspective of entrepreneurial psychology, enterprises are essential to maximize profits, that is, to maximize values. Therefore, the significance of this study on CSR is actually the study of CSR value creation (Ding and Jin, 2020).

### Analysis of Corporate Reputation

Corporate reputation is an attribute to the enterprise, and it can be deduced from the enterprise behavior. It is also known as the belief of enterprise strategic characteristics. Corporate reputation is the process of changing the public’s cognitive psychology, and is the sum of the ability of corporate behavior to obtain social recognition, thereby obtaining resources, opportunities, and support, and then completing value creation (Adelua et al., 2021). According to the definition given by foreign psychological researchers, the definition of the cognitive domain from the perspective of entrepreneurial psychology is extended, and it contains not only the objective cognition but also the subjective cognition. Therefore, the evaluation of corporate reputation comes from the subjective cognition of the attributes of a company, as well as the views on the inherent tendencies of these attributes. From the perspective of entrepreneurial psychology, enterprises naturally hope that corporate reputation can be evaluated from a multidimensional subjective and objective perspective rather than a biased examination (Tia et al., 2021).

### Factors Influencing Corporate Social Responsibility

Taking social responsibility can make enterprises gain a competitive advantage and promote innovation and global economic growth, and create value from the perspective of entrepreneurial psychology (Ritala et al., 2021). CSR is related to the characteristics of corporate ownership, country, and cultural background. There are great differences in CSR between family firms and non-family firms. Enterprises with large scale, high earnings per share after deduction, and large number of independent directors are more willing to actively disclose the fulfillment of CSR. The lower the degree of government intervention in the economy is, the better the legal environment and the more developed the factor market is, and the better the CSR is taken. The fulfillment of CSR is related to the reputation and social impact of enterprises. The voluntary disclosure level of enterprises with good social reputation is high but not significant. The voluntary disclosure level of enterprises with poor social reputation is significantly low. The development of information technology affects the fulfillment of CSR and the level of information disclosure (Negash et al., 2021). Whether or not an enterprise undertakes social responsibility, what kind of social responsibility it undertakes, and how much social responsibility it undertakes are determined by various circumstances. CSR is affected not only by a country’s history, culture, system, and economic development level but also by various internal and external factors such as developed countries, multinational companies, the company’s own scale, ownership, and expected psychology. These factors affect and limit the scope, quantity, and types of CSR. Internal corporate governance is an important factor affecting CSR.

The role and importance of social media in society become increasingly prominent, and the influence caused by its participants is huge. Enterprises also pay more attention to the application of social media because it is a new way to communicate with customers and support internal communication and collaboration. How to use social media to bring value to enterprises becomes one of the important strategies of enterprises (Hao et al., 2021).

### Mechanism of Social Media on Corporate Social Responsibility

With the popularization and application of social media, the communication medium of CSR changes, and the path of CSR to create value is affected from the perspective of entrepreneur psychology. Enterprise managers should adapt to the change in the environment to better serve the value creation of CSR.

(1) The “platform” of social media makes the value creation of social responsibility a reality.

The value creation of CSR mainly includes the cultural construction path, social capital accumulation path, and reputation promotion path (Gillan et al., 2021). In fact, the communication channel between enterprises and stakeholders is one-way, and it is difficult for enterprises to obtain valuable feedback information before the emergence of social media. Social media provide platforms for communication between enterprises and stakeholders, making it possible to construct corporate culture, accumulate social capital, and enhance corporate reputation.

(2) Dialogue of social media leads to CSR value creation.

With the help of social media, stakeholders have the information about CSR activities, which provides a solid foundation for accumulating social capital, building corporate culture, and enhancing corporate reputation. Besides, it enables enterprises to master the “demand information” of stakeholders on CSR, which provides a prerequisite for enterprises to implement CSR activities in a targeted manner (Liu N. et al., 2020).

(3) Communicability of social media enhances CSR value creation.

The value creation ability of CSR is ultimately determined by the communication effect of information. Compared with traditional media, social media speed up the interpersonal communication, improve the effect of organizational communication, and enhance the influence of mass communication (Montag et al., 2021).

First, social media enables people to communicate through the behavior of CSR anytime and anywhere, thereby accelerating the communication of social responsibility between people. Second, social media transmits social responsibility more smoothly to organization members. Through top-down information transmission, corporate managers can establish “WeChat groups,” “QQ groups,” video accounts, official accounts, “Weibo accounts,” and other methods to convey short-term or long-term CSR. Therefore, through the comparison between different social media, the main influence of different social media on CSR communication is studied.

### The Hypotheses Proposed

Corporate social responsibility fulfillment is an opportunity for enterprises. Enterprises should face the concerns of the whole society by providing new technologies, new methods, and innovative management methods to create shared values for enterprises and society (Grygiel and Brown, 2019). CSR performance has a positive impact on corporate reputation. Only when relevant stakeholders realize that enterprises are responsible for their behavior they can form a positive evaluation of enterprises and promote the establishment of corporate reputation. The emergence of social media provides the possibility for this two-way interaction.

Among the listed companies, the immediacy and coverage of social media mean that enterprises can no longer hide behaviors that may have adverse effects, which forces enterprises to manage CSR in a more realistic way and be responsible for ordinary citizens (Zhao et al., 2022). Social media have a great potential for the communication of CSR and also help enterprises to sort out good social images for different stakeholders (Arslan and Wong, 2022). Although the popularity of social media is high, it is unknown whether the initiative and enthusiasm of enterprises for the use of social media affect CSR evaluation, and whether the interaction between social media and stakeholders helps improve CSR scores. In this case, the first research hypothesis is put forward.

Hypothesis 1: The opening of social media of the listed companies is positively correlated with the increase in CSR scores.

The emergence of social media provides a two-way channel for the public to know about CSR information. Does user interaction on social media have a negative effect on information disclosure and social reputation of the listed companies? Specifically, will social media users’ reading and interaction affect the performance of CSR and information disclosure of the listed companies after CSR is taken? Will users’ attention and support improve their CSR scores? The second hypothesis is put forward to explore the influence of user reading and likes on CSR scores.

Hypothesis 2: The number of users’ reading and likes of specific CSR is positively correlated with the increase in CSR score of the listed companies.

In China, the largest social media networks are “micro-blog” or “WeChat.” User attributes, social relations, and “micro-blog” are the three major factors affecting user forwarding behavior. Based on user forwarding behavior, it is found that “micro-blog” is a social platform, and its users’ social needs are much higher than their needs. With the advent of the Internet era, “WeChat” is the most typical application in this era. It integrates “instantaneity” and “socialization,” showing the development trend of the future. The emergence of “WeChat public number” establishes an effective channel for enterprises to communicate with users and other stakeholders. The social media platforms ranked after “WeChat” include QQ, micro-blog, “Zhihu,” and “Douban.” From the market share of social media, the third hypothesis is put forward based on users’ number.

Hypothesis 3: Different social media have different impacts on CSR communication. Among them, the WeChat platform performs better than other social media, so it can establish a stable corporate reputation for itself.

### Sample Selection and Research Design

A total of 78 listed companies in the mobile phone industry before 1 August 2020, according to *The industry classification results of city companies in the first half of 2020* published on the CSR as the research object, combined with Record Keeping Server (RKS) social responsibility rating database of social responsibility score the relationship between CSR information disclosure of social network channels and CSR score.

The reason why the mobile phone industry is chosen is as follows: (1) the public’s perception of the mobile phone industry is stronger and (2) the mobile phone is an indispensable industry in life, which is always connected with the public. It is possible to get more attention on social networks.

Before 1 August 2020, there are 60 companies with “social media account” in 78 companies. The observation data of CSR information disclosure based on social network come from the information released by 60 company samples during the period from 1 August 2010 to 31 December 2020. Among the data used in this study, the publication time, title, the number of readings, and “likes” are obtained manually, and the number of readings and “likes” is the data as of 31 December 2020. The measurement tool used in this study is SPSS 25.00.

In this study, the user data on social media background is mainly collected through online questionnaires. In the early stage, the social media accounts of all sample companies are concerned, and the media operation departments of each company are fans. The questionnaire is distributed from September 2020 to October 2020. A total of 150 questionnaires are distributed and 130 questionnaires are retrieved with a recovery rate of 86%.

## Research Results of the Influence of Social Media on Corporate Social Responsibility Communication

### Effects of Different Social Media Platforms on Corporate Social Responsibility Communication

**FIGURE 2:**
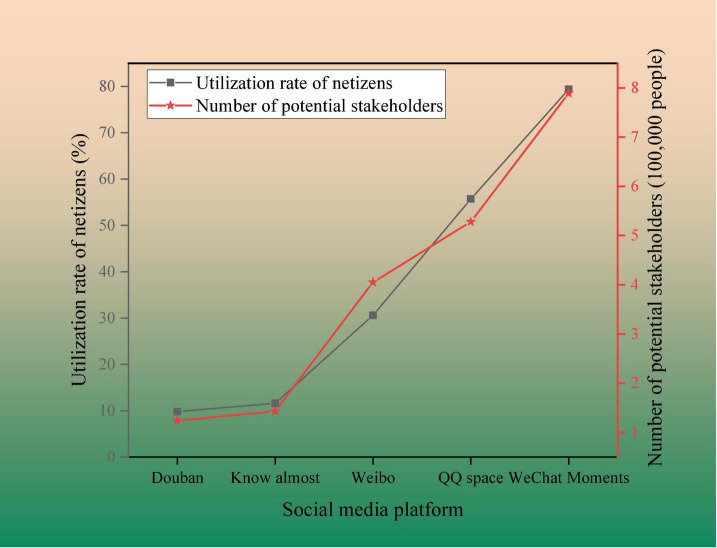
Impact of different social media account platforms on CSR communication.

The social media accounts of the listed companies in the mobile phone industry are counted. The influence of different social media account platforms on CSR is shown in Figure 2.

In Figure 2, among them, different social media platforms include “Douban, Zhihu, Weibo, Qzone, and WeChat groups.” The online questionnaire survey shows that companies using “WeChat” to acquire customers have low cost, large user base, and traffic flow. There are many “daily activities,” and the level of interaction is much higher than other social media. The “WeChat” platform has the highest proportion of potential customers who read and like it, reaching 800,000. The ratio is close to 80%. This proves that hypothesis 3 is valid, that is, different social media have different effects on CSR communication.

**FIGURE 3:**
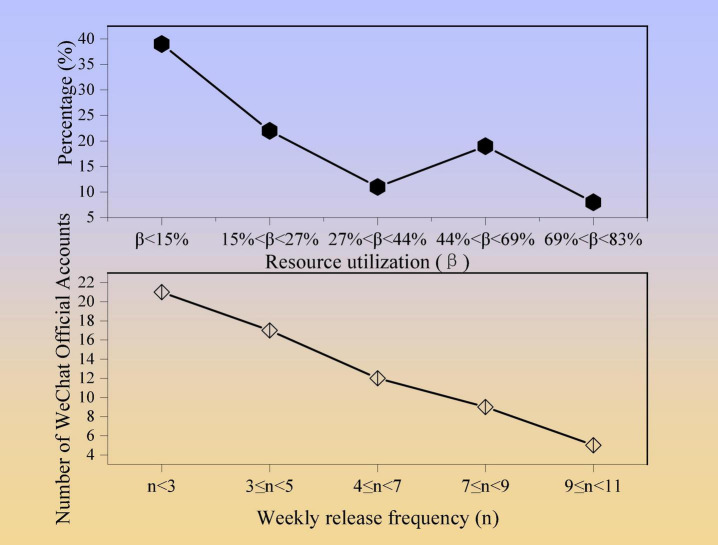
Resource utilization and frequency statistics of CSR communication on WeChat.

Further analysis of 60 companies that open “WeChat” can obtain the utilization rate and frequency of information released by enterprises. The resource utilization and frequency of CSR communication by sample companies using “WeChat” are shown in Figure 3.

According to statistics, there are 21 companies that publish social media less than three times a week. There are 17 companies that publish 3–5 times. Only five public accounts (12%) publish graphic information more than nine times a week. These public accounts have fully utilized the push frequency resources of WeChat. In general, the average number of samples released per week is 6.1.

### Analysis of the Opening of Social Media and the Increase of Corporate Social Responsibility Score of Listed Companies

**FIGURE 4:**
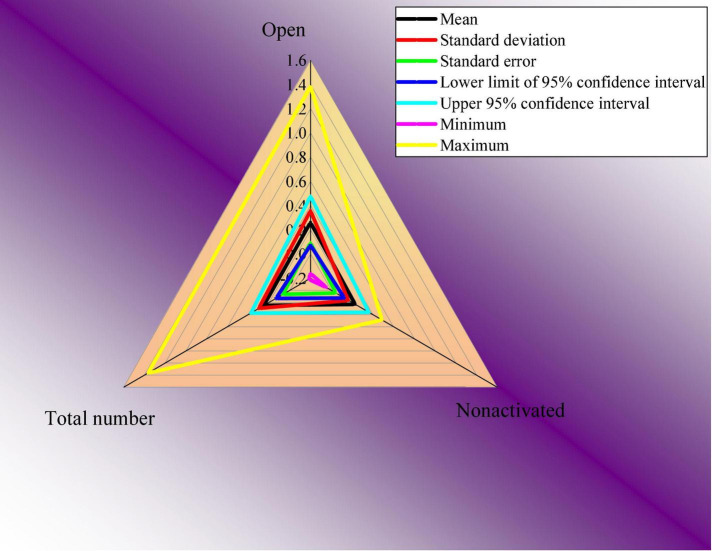
Results of descriptive statistics.

According to the statistical data of the questionnaire, the SPSS 25.00 data analysis software is used to analyze the influence of the opening of the social media of the sample enterprises on the annual score of CSR. Because the radar chart can comprehensively analyze multiple indicators, it has the advantages of being complete, clear, and intuitive. Therefore, the results are plotted as a radar chart. Descriptive statistical analysis is conducted, and the results are shown in Figure 4.

**FIGURE 5:**
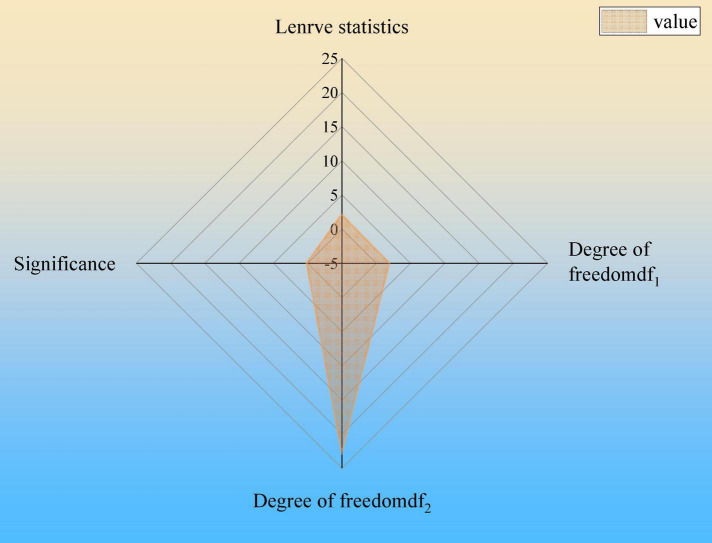
Variance homogeneity test.

In Figure 4, descriptive statistics are analyzed from seven dimensions, namely, mean, standard deviation, standard error, maximum value, minimum value, the lower limit of the 95% confidence interval, and the upper limit of the 95% confidence interval. The analysis shows that the significance of the homogeneity test of variance is 0.158, which is greater than 0.05, and variance analysis can be carried out. The homogeneity test results are shown in Figure 5.

**FIGURE 6:**
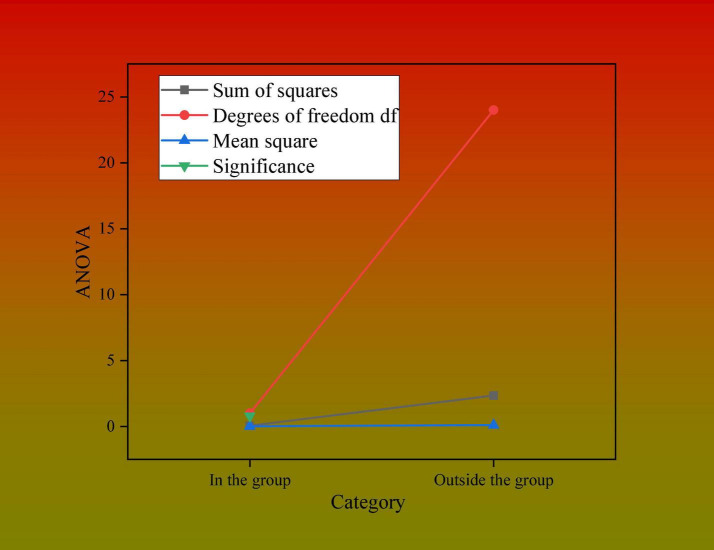
One-way analysis of variance.

The total sample in the figure is 25, because only 25 companies have published CSR score data. The results of variance analysis are shown in Figure 6.

The significance of variance analysis is 0.804, which proves that whether to open the social media account has no significant difference in the growth of the CSR score. The above hypothesis cannot be verified. The opening of social media has an impact on the CSR score of listed companies through new media operations and user reading and interaction. In this regard, hypothesis 1 is not true. The openness of social media in listed companies is not significantly correlated with the improvement of CSR scores.

### Statistical Analysis Results of User Reading, Likes, and Corporate Social Responsibility Scores

A total of 13 listed companies are selected according to CSR scores between 2019 and 2020 from 60 samples of social media, and the correlation between the user reading of media and the increase in CSR score is analyzed. First, the reading and likes are standardized, and then Pearson correlation analysis is performed. The results are shown in Table 1.

**TABLE 1:** Pearson correlation test of potential customers’ readings, likes, and CSR scores.

	Reading	Likes	Compared with the increase in 2020
Reading	1	0.937**	0.387
Likes	0.937**	1	0.534*
Compared with the increase in 2020	0.387	0.534*	1

*Represents a significant correlation at 5% level (bilateral).

**Represents a significant correlation at 5% level (bilateral).

The results manifest that the number of user reading and likes is not significantly correlated with the increase in CSR score, while user reading is significant compared with 2020, and the rest are not significant. However, the information disclosed by social media will indeed have an impact on the communication of CSR. For this reason, a correlation test is further carried out on the relationship chain of shareholders and other closely related enterprises. The test results are shown in Table 2.

**TABLE 2:** Pearson correlation test of CSR reading related to corporate shareholders, number of likes, and CSR.

	Number of readings	Number of likes	Score increase
Number of readings	1	0.913**	0.564*
Number of likes	0.913**	1	0.655
Score increase	0.564	0.655	1

*Indicates significant correlation at 5% level (bilateral).

**Indicates significant correlation at 5% level (bilateral).

**FIGURE 7:**
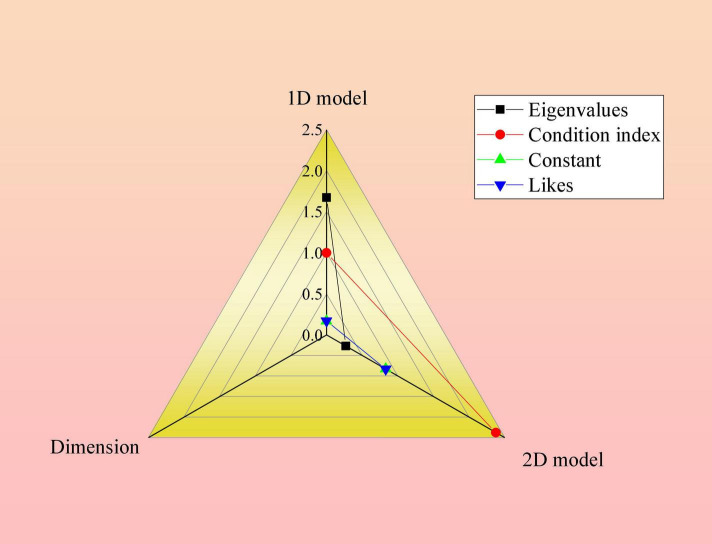
Collinearity diagnostic diagram of the linear regression test between the number of likes of social media associated with the content and the growth rate of CSR scores. In the figure, the dependent variable of the scale is the growth rate of the score, and the predictive variable is the number of likes.

In Table 2, in the Pearson correlation test between the number of CSR readings and the company’s shareholders, the number of likes, and CSR, the analysis results of users’ reading and likes on these two types of content found that the number of CSR user likes related to shareholders is significantly correlated with the increase of CSR score, and the rest are not significantly correlated. The results are tested for linear regression to verify the correlation. The collinearity diagnosis of the linear regression test is shown in Figure 7.

**FIGURE 8:**
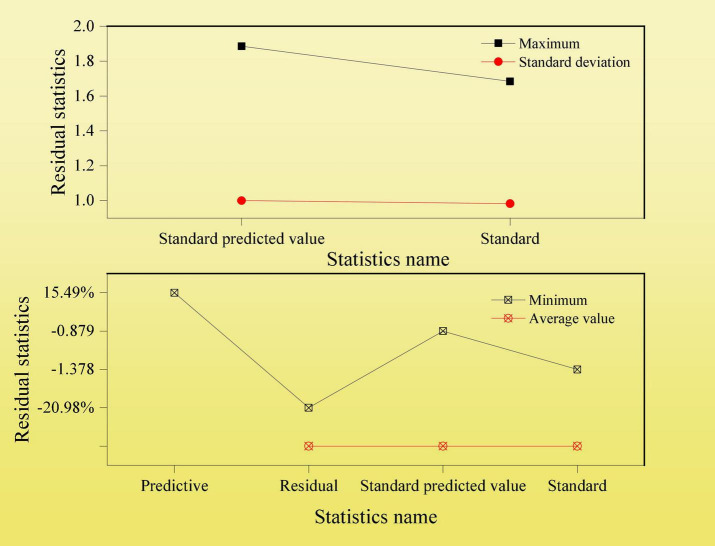
Residual statistical indexes of the linear regression test between the content of likes and CSR score growth rate.

The residual statistical diagram of the linear regression test between the number of likes related to the content and the growth rate of CSR scores is shown in Figure 8.

According to the results of the above linear regression test, the variable linear regression coefficient is less than 0.05, indicating that the number of likes related to the content is significantly related to the increase of CSR scores. Therefore, hypothesis 2 is true. The number of users who read and like a particular CSR is positively correlated with an increase in the listed company’s CSR score. Therefore, companies need to analyze and improve their CSR scores, such as by posting content related to shareholder interests on social media.

To sum up, from the perspective of entrepreneurial psychology, different social media platforms have different effects on the communication of CSR. “WeChat” is relatively good for the communication of CSR. The proportion of potential customers who read and like the company’s dynamic display through the “WeChat” platform is the highest. It can reach up to 800,000 among the surveyed sample companies. WeChat communication can bring impetus to the creation of CSR value and continuously improve the reputation of enterprises. There is no positive correlation between the openness of social media and the improvement of CSR scores of listed companies, but it is necessary to properly operate social media, so that the value of entrepreneurial psychology can be maximized. User readings and likes are positively correlated with the increase in CSR score. The results show that the linear regression coefficient between the number of likes and the increase in CSR score is less than 0.05. Previous literature has studied the influencing factors and economic consequences of CSR, and it is conducive to improve corporate performance. Different types of social media have different impacts on the communication of CSR, and the value creation of CSR is also different. Therefore, companies need to analyze and improve their CSR scores, such as by posting content related to shareholder interests on social media. For ordinary customers, it is possible to improve communication in terms of customer forwarding behavior, the number of comments, customer stickiness, and traffic statistics to promote the communication of CSR, and to make the level of corporate reputation more stable and better for long-term development.

## Conclusion

At present, the development of the Internet has given birth to social media such as Weibo and WeChat. Companies use social media to take CSR, explore the relationship between current customers and other stakeholders, and thus enhance their reputation. The influencing factors of CSR performance from a macro perspective are expounded, and the role of social media in CSR is briefly discussed. Hypotheses are formulated and validated. The following conclusions are drawn: (1) different types of social media have different effects on CSR communication, and the value creation of CSR is also different; (2) among many social media, WeChat has the greatest impact on CSR and can provide the impetus for the improvement of corporate reputation; (3) the opening of social media is not directly related to the improvement of CSR scores; (4) only the correct operation of social media will have an impact on the communication of CSR; and (5) the correlation between users’ reading and likes on social media and the increase of CSR score is not significant. Only the number of likes on content-related social media is significantly related to the increase in CSR score. All in all, first, the WeChat platform has the best communication effect on CSR, which can provide the impetus for the improvement of corporate reputation. Second, the opening of social media is not directly related to the improvement of CSR scores, but the correct operation of social media will have an impact on the communication of CSR. Finally, only the number of likes on shareholder-related social media is significantly associated with an increase in the CSR score. Therefore, companies need to analyze and improve their CSR scores, such as by posting content related to shareholder interests on social media. For ordinary customers, it is possible to improve communication in terms of customer forwarding behavior, number of comments, customer stickiness, and traffic statistics, in order to promote the communication of CSR, and to make the level of corporate reputation more stable and better for long-term development. Although the research follows the laws of the experimental process, it is inevitable that there are some deficiencies caused by subjective and objective factors. For example, differences between samples from different industries are not considered. Macro factors, such as the regionality of the sample companies, the economic development level of the sample locations, and the social media development level of the sample companies, have not been demonstrated in detail. Therefore, research in this area will be strengthened in the future, and the role of social media in the communication of CSR will be more prominent.

## Data Availability Statement

The raw data supporting the conclusions of this article will be made available by the authors, without undue reservation.

## Ethics Statement

The studies involving human participants were reviewed and approved by the University of Amsterdam Ethics Committee. The patients/participants provided their written informed consent to participate in this study. Written informed consent was obtained from the individual(s) for the publication of any potentially identifiable images or data included in this article.

## Author Contributions

The author confirms being the sole contributor of this work and has approved it for publication.

## Conflict of Interest

The author declares that the research was conducted in the absence of any commercial or financial relationships that could be construed as a potential conflict of interest.

## Publisher’s Note

All claims expressed in this article are solely those of the authors and do not necessarily represent those of their affiliated organizations, or those of the publisher, the editors and the reviewers. Any product that may be evaluated in this article, or claim that may be made by its manufacturer, is not guaranteed or endorsed by the publisher.
